# Potentiating the Anti-Tuberculosis Efficacy of Peptide Nucleic Acids through Combinations with Permeabilizing Drugs

**DOI:** 10.1128/spectrum.01262-21

**Published:** 2022-02-16

**Authors:** Karishma Berta Cotta, Saptarshi Ghosh, Sarika Mehra

**Affiliations:** a Centre for Research in Nanotechnology and Science, Indian Institute of Technology Bombay, Mumbai, Maharashtra, India; b Wadhwani Research Centre for Bioengineering, Indian Institute of Technology Bombay, Mumbai, Maharashtra, India; c Department of Chemical Engineering, Indian Institute of Technology Bombay, Mumbai, Maharashtra, India; Indian Institute of Science Bangalore

**Keywords:** ethambutol, peptide nucleic acids, permeability, tuberculosis, intramacrophage

## Abstract

The emergence of antimicrobial resistance warrants for the development of improved treatment approaches. In this regard, peptide nucleic acids (PNAs) have shown great promise, exhibiting antibiotic properties through the targeting of cellular nucleic acids. We aimed to study the efficacy of PNA as an anti-tuberculosis agent. Since the efficacy of PNA is limited by its low penetration into the cell, we also investigated combinatorial treatments using permeabilizing drugs to improve PNA efficacy. Various concentrations of anti-*inhA* PNA, permeabilizing drugs, and their combinations were screened against extracellular and intracellular mycobacteria.0.625 to 5 μM anti-*inhA* PNA was observed to merely inhibit the growth of extracellular M. smegmatis, while low intracellular bacterial load was reduced by 2 or 2.5 log-fold when treated with 2.5 or 5 μM PNA, respectively. Anti-*inhA* PNA against M. tuberculosis H37Ra exhibited bactericidal properties at 2.5 and 5 μM and enabled a slight reduction in intracellular M. tuberculosis at concentrations from 2.5 to 20 μM. Of the permeabilizing drugs tested, ethambutol showed the most permeabilizing potential and ultimately potentiated anti-*inhA* PNA to the greatest extent, reducing its efficacious concentration to 1.25 μM against both M. smegmatis and M. tuberculosis. Furthermore, an enhanced clearance of 1.3 log-fold was observed for ethambutol-anti-*inhA* PNA combinations against intracellular M. tuberculosis. Thus, permeabilizing drug-PNA combinations indeed exhibit improved efficacies. We therefore propose that anti-*inhA* PNA could improve therapy even when applied in minute doses as an addition to the current anti-tuberculosis drug regimen.

**IMPORTANCE** Peptide nucleic acids have great potential in therapeutics as anti-gene/anti-sense agents. However, their limited uptake in cells has curtailed their widespread application. Through this study, we explore a PNA-drug combinatorial strategy to improve the efficacy of PNAs and reduce their effective concentrations. This work also focuses on improving tuberculosis treatment, which is hindered by the emergence of antimicrobial-resistant strains of Mycobacterium tuberculosis. It is observed that the antibacterial efficacy of anti-*inhA* PNA is enhanced when it is combined with permeabilizing drugs, particularly ethambutol. This indicates that the addition of even small concentrations of anti-*inhA* PNA to the current TB regimen could potentiate their therapeutic efficiency. We hypothesize that this system would also overcome isoniazid resistance, since the resistance mutations lie outside the designed anti-*inhA* PNA target site.

## INTRODUCTION

Through their anti-gene/anti-sense properties (1–4), peptide nucleic acids (PNAs) display antibacterial activity against many pathogenic bacteria, such as Escherichia coli (5–7), Klebsiella pneumonia (8), Pseudomonas aeruginosa (9), Salmonella enterica (10, 11), Staphylococcus aureus (12), and Brucella suis (13) when targeting essential genes like *gyrA* (8, 12), *ftsZ* (4, 9), *acpP* (9, 14), and *rpoB* (13). Their application in therapeutics, however, is mainly limited by the low penetration capacity of PNA across cell membranes (3, 15), particularly bacterial membranes (16). PNA encapsulation in nanoparticles (17), linkage to antimicrobial peptides (18), and conjugation to cell penetrating peptides (CPPs) (5) have been explored to improve its uptake. PNA-CPP conjugates demonstrate 15- to 20-fold greater efficacy than PNA alone (14). The efficacious concentrations, however, remain high.

Treatment of *in vitro* and *in vivo* bacterial infections caused by permeable mutants of E. coli demonstrates a lower dosage requirement of PNA/PNA-CPP compared to treatment of infections with wild type (WT) strains (7, 14, 19). Combinations of PNA with a nonapeptide or conjugated to CPP prove to enhance efficacy only partially in WT strains, where permeable mutants continue to exhibit greater susceptibility (7, 19). Thus, a combinatorial therapy of CPP-PNA with a permeabilizing drug could enhance overall bactericidal efficacy.

Our study focuses on the evaluation of a PNA-CPP targeting an essential gene for treatment of tuberculosis (TB). Kulyté et al. (2005) demonstrated that anti-*inhA* PNA targeting the gene start site in Mycobacterium smegmatis could partially inhibit its growth at 5 μM over a 16-h period (20). We investigated the effect of permeabilizing drugs, colistin, ceftazidime pentahydrate, or ethambutol, on the efficacy of this anti-*inhA* PNA. Colistin, ceftazidime, and ethambutol target cations in the lipopolysaccharide layer (21), penicillin-binding proteins (22), and arabinogalactan/lipoarabinogalactan synthesis (23), respectively. Colistin and ethambutol have additionally been found to improve the efficacy of other anti-TB drugs (24, 25) while combinations of ceftazidime and avibactam are effective against the Mycobacterium avium complex (26). Thus, the permeabilizing activity of these drugs was hypothesized to improve the efficacy of anti-*inhA* PNA by enhancing PNA uptake.

We also test the efficacy of anti-*inhA* PNA designed to target the start site of the *inhA* in M. tuberculosis and examined its efficacy in combination with permeabilizing drugs against both extracellular and intramacrophage mycobacteria.

## RESULTS

Through the targeting of an essential gene, it has been proven that peptide nucleic acids (PNAs) can have antibacterial effects (27–30). *InhA*, encoding an NADPH-dependent enoyl-acyl carrier protein reductase, is an essential gene for the synthesis of the fatty acids that constitute the mycolic acid in the mycobacterial cell wall (31). *InhA* inhibition or downregulation is known to effectively inhibit survival of Mycobacterium (32, 33). Thus, *inhA* was selected for our study. Anti-*inhA* PNA designed against M. smegmatis has a growth-inhibitory effect against this bacterium. However, efficacy was observed to be achieved at concentrations of no less than 5 μM (20). We selected this predesigned anti-*inhA* PNA for our study and also designed an anti*-inhA* PNA against M. tuberculosis (Table 1).

**TABLE 1:** Anti-*inhA* PNA sequences used in this study to target either M. smegmatis or M. tuberculosis

CPP-PNA sequence	Bacterial target	Size (bp)	Reference
(KFF)_3_K-GTCATTTGGT-NH_2_	*inhA* M. smegmatis	10	(20)
(KFF)_3_K-CTGTCATGTGCG-NH_2_	*inhA* M. tuberculosis	12	This study
(KFF)_3_K-GTGTCATGTGCG-NH_2_	Mismatch M. tuberculosis	12	This study

### Anti-mycobacterial effect of anti-*inhA* PNA and permeabilizing agents against M. smegmatis.

**FIG 1:**
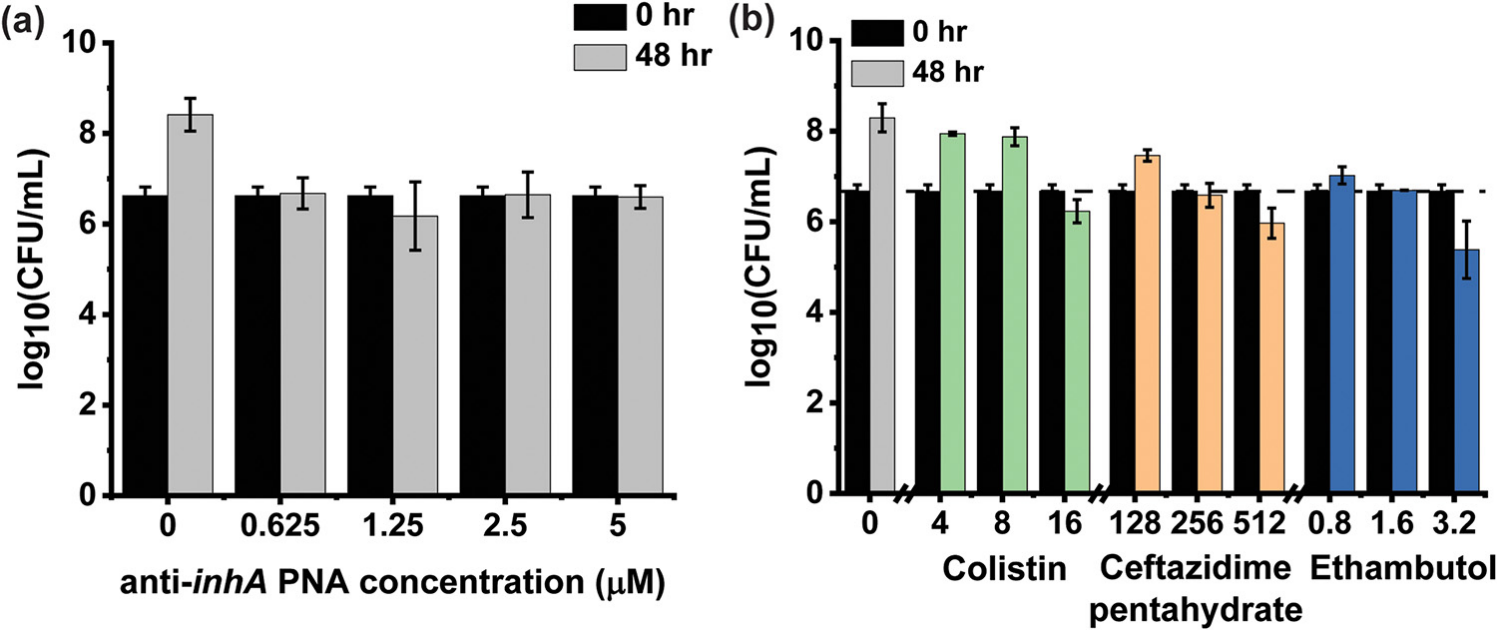
Effects of increasing concentrations of (a) anti-*inhA* PNA and (b) permeabilizing drugs (colistin, ceftazidime pentahydrate, and ethambutol) on the survival of M. smegmatis after 48 h at 37°C. The data are plotted from 5 biological replicates and error bars indicate the standard deviation for each sample.

The treatment of 10^6^ CFU/mL of M. smegmatis with 0.625 to 5 μM of anti-*inhA* PNA over 48 h was observed to have growth-inhibitory effects, maintaining a constant cell density throughout the treatment time (Fig. 1a). This was consistent with the reports by Kulyté et al. (2005) which demonstrated that 2 and 5 μM anti-*inhA* PNA displayed similar growth inhibitory profiles at 16 h (20).

To facilitate the entry, and therefore the efficacy, of the anti-*inhA* PNA, we propose a combinatorial treatment with a permeabilizing drug. The MICs of colistin, ceftazidime pentahydrate, and ethambutol were therefore determined. Colistin was found to have an MIC of 16 mg/L, while ceftazidime pentahydrate and ethambutol exhibited MICs of 256 mg/L and 1.6 mg/L, respectively (Fig. 1b). It is expected that the bacteriostatic concentrations of these drugs would be optimal for altering cell membrane permeability without causing cell death.

### Effect of permeabilizing drugs on membrane permeability of *M. smegmatis*.

**FIG 2:**
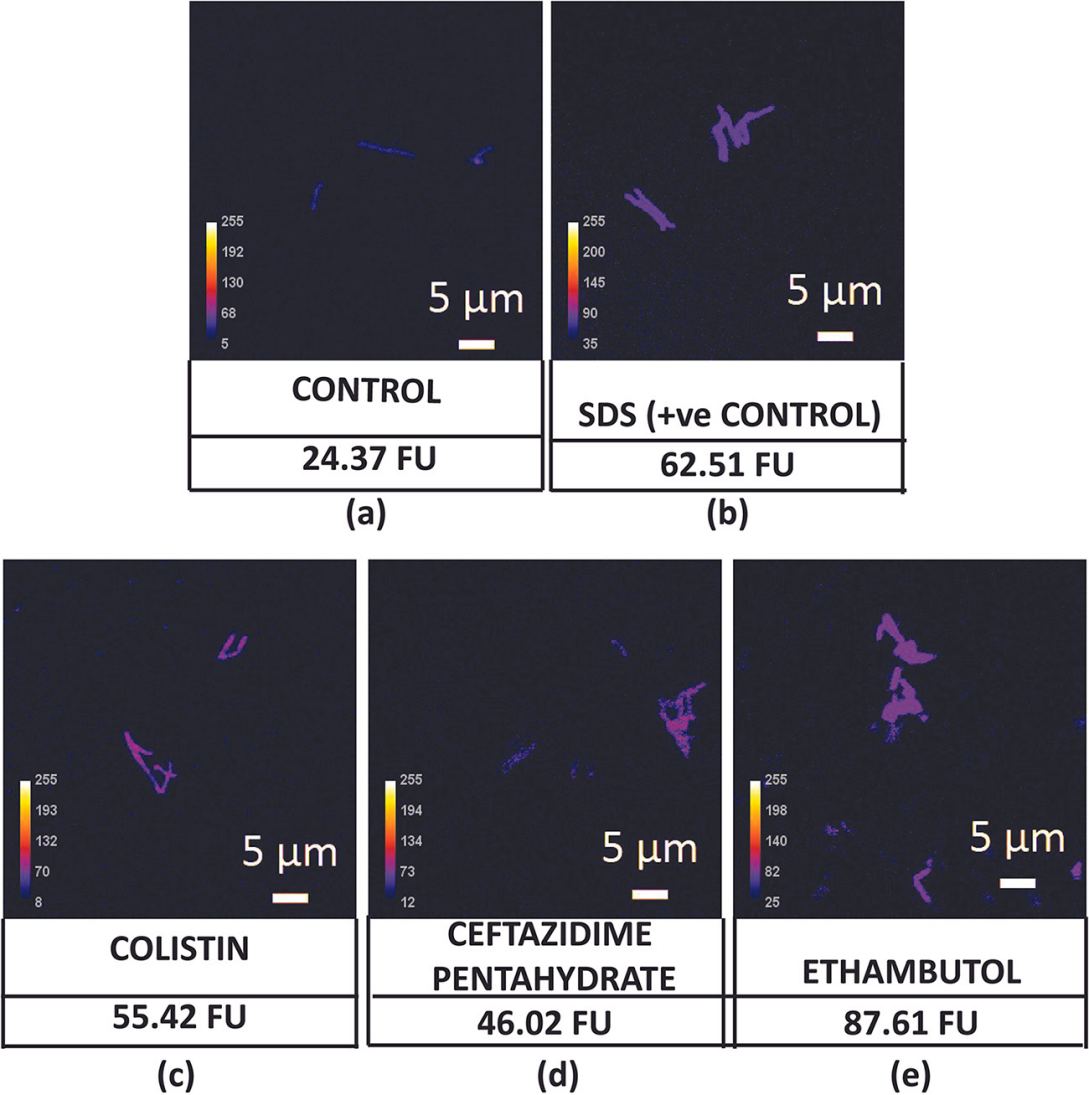
Analysis of the effects of permeabilizing drugs at their MICs on the membrane permeability of M. smegmatis at 4 h. Confocal imaging of (a) nontreated control, (b) SDS-treated control, and (c) colistin-, (d) ceftazidime pentahydrate-, and (e) ethambutol-treated M. smegmatis stained with NPN dye. FU (fluorescence units) indicates corrected total cell fluorescence.

To confirm the permeabilization of M. smegmatis cells treated with inhibitory concentrations of colistin, ceftazidime pentahydrate, and ethambutol, an NPN assay was performed. The hydrophobic dye NPN (1-*N*-phenylnapthylamine) exhibits low fluorescence in an aqueous environment. Entry into lipid-rich cell membrane causes an increase in fluorescence intensity. Any alteration of cell membrane integrity increases entry of the dye via the cell membrane, resulting in increased fluorescence (34). Confocal imaging of NPN-stained cells after 4 h of permeabilizing drug treatment showed that these cells had a higher fluorescence compared to the untreated control (Fig. 2), confirming that the treatment enhanced the permeability of M. smegmatis cells. Note that cell density prior to NPN staining was maintained constantly across all samples.

### Combinatorial effect of permeabilizing drugs and various anti-*inhA* PNA concentrations.

**FIG 3:**
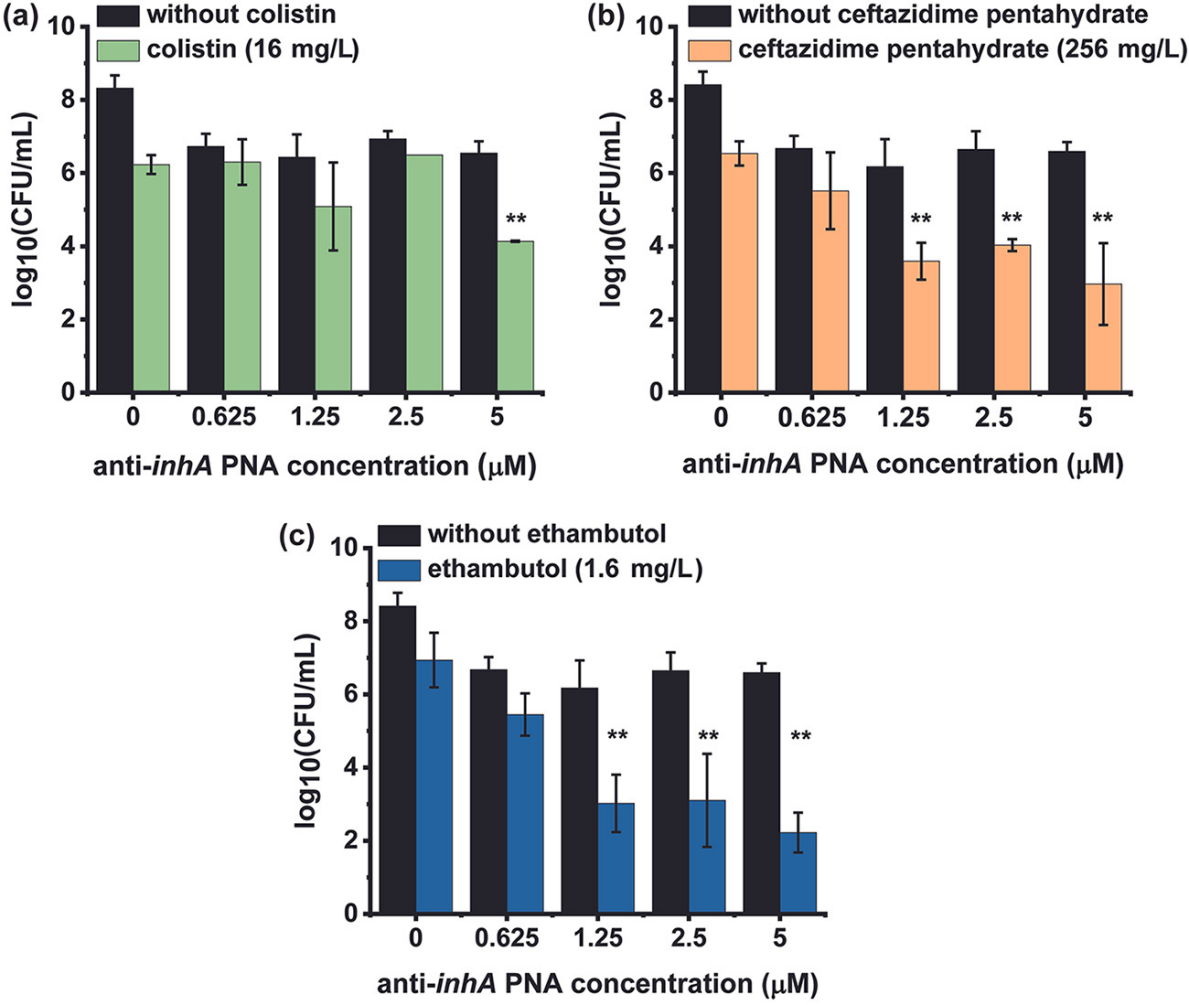
Combinatorial effects of permeabilizing drugs and anti-*inhA* PNA against M. smegmatis. The efficacy of (a) colistin, (b) ceftazidime pentahydrate, or (c) ethambutol in combination with 0.625 to 5 μM anti-*inhA* PNA after 48 h treatment at 37°C. Data plotted are obtained from a sample size of *n* = 3, where error bars indicate the standard deviation for each sample. Significance is indicated by ** for *P*-value ≤ 0.01 as determined through Tukey-Kramer *post hoc* test for combinatorial treatments with respect to corresponding drug-alone and PNA-alone treatments.

MICs of each drug were combined with incrementally increasing concentrations of anti-*inhA* PNA. The combined efficacy was determined after 48 h of treatment. Treatment with 16 mg/L of colistin improved the efficacy of 5 μM anti-*inhA* PNA by 2.5 log-fold. At this concentration, however, colistin had no effect on the efficacy of 0.625 to 2.5 μM anti-*inhA* PNA (Fig. 3a). Treatment with 256 mg/L of ceftazidime pentahydrate in combination with 1.25, 2.5, and 5 μM anti-*inhA* PNA resulted in 2.6, 2.6, and 3.6 log-fold reductions in bacterial load, respectively (Fig. 3b). Ethambutol, which demonstrated the highest effect on membrane permeability in the NPN assay, enhanced the efficacy of 1.25 to 5 μM anti-*inhA* PNA, resulting in a 3 to 4 log-fold greater reduction in bacterial load (Fig. 3c). Thus, permeabilizing drugs do improve the efficacy of anti-*inhA* PNA, and the extent of this increase could depend on the drug’s mode of action and/or its efficiency in disrupting the bacterial membrane.

Since the anti-*inhA* PNA targets the same cellular process as the anti-TB drug isoniazid (INH), we also investigated the combinatorial efficacy of permeabilizing drugs with INH through a disk diffusion assay. We used a disk diffusion assay here since INH in liquid culture (Middlebrook 7H9 broth) did not exhibit an MIC against M. smegmatis up to concentrations as high as 1024 mg/L. In this study, colistin, ceftazidime pentahydrate, and ethambutol were loaded, individually or in combination with INH, on sterile discs placed on LB agar spread-plated with M. smegmatis culture. The clearance zone indicated the efficacy of the loaded antibiotic(s). We observed increased efficacy only for combinatorial treatments of INH (32 μg) with ceftazidime pentahydrate (512 and 1024 μg), where inhibition zone diameters increased from ≤10 mm to 13.5 to 14.5 mm.

### Toxicity of permeabilizing drugs to macrophage cells.

Translation of this combinatorial therapy for treatment of intracellular pathogens requires that the compounds exert minimal toxicity toward the host. The toxicity of the permeabilizing drugs toward the macrophage cells was therefore monitored over 48 h. At 48 h, colistin exhibited no toxicity at concentrations up to 32 mg/L. Macrophage viability was reduced to 22% on treatment with 128 mg/L of colistin (data not shown). Ceftazidime pentahydrate exhibits a slight toxicity at 1024 mg/L over 48 h, where the relative viability dropped to 65% (data not shown). Treatment of 4 to 16 mg/L of ethambutol was found to be nontoxic over 48 h (data not shown). Thus, colistin is considered to be nontoxic at concentrations of ≤64 mg/L, ceftazidime pentahydrate exerts a toxicity at concentrations of ≥1024 mg/L, and ethambutol is nontoxic at concentrations of ≤16 mg/L.

### Effect of anti-*inhA* PNA and cell wall-targeting drug-PNA combinations on the survival of intracellular *M. smegmatis*.

**FIG 4:**
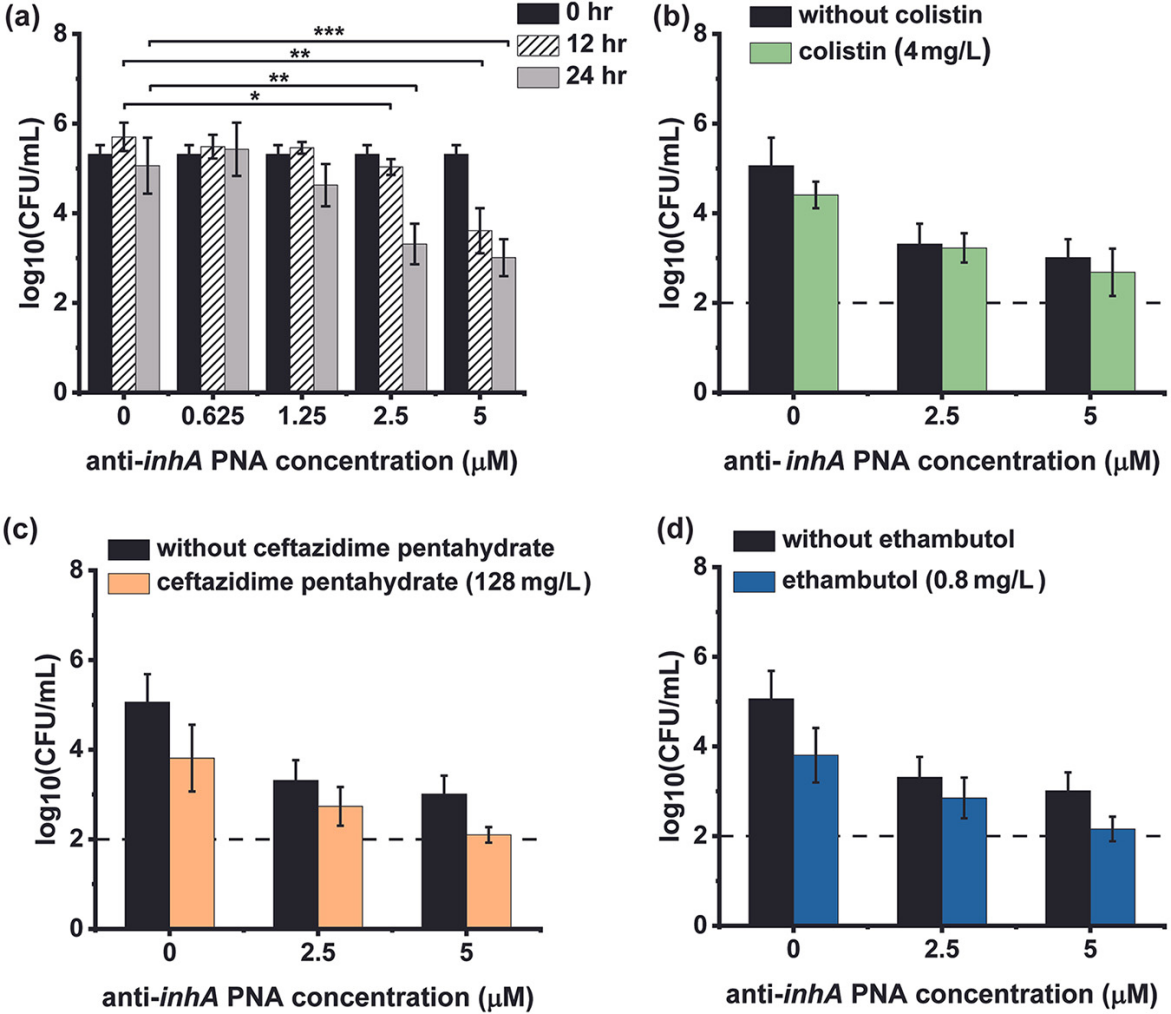
Efficacy of anti-*inhA* PNA alone and in combination with permeabilizing drugs against intramacrophage M. smegmatis. Intracellular bacterial load post treatment with (a) anti-*inhA* PNA alone over 12 and 24 h and (b) colistin, (c) ceftazidime pentahydrate, and (d) ethambutol combined with 0 to 5 μM anti-*inhA* PNA over 24 h. Data were obtained from 3 biological replicates and error bars indicate the standard deviation of each sample. Significance is indicated by * for *P*-value ≤ 0.05, ** for *P*-value ≤ 0.01, and *** for *P*-value ≤ 0.001.

To evaluate the efficacy of anti-*inhA* against intramacrophage M. smegmatis, we infected differentiated THP-1 cells with M. smegmatis. The intracellular bacterial load achieved was 10^5^ CFU/mL. Treatment of intracellular M. smegmatis cells with anti-*inhA* PNA resulted in a time- and concentration-dependent reduction in intracellular bacterial load. The highest concentration tested, 5 μM anti-*inhA* PNA, resulted in 1.5 and 2.5 log-fold reductions in intramacrophage M. smegmatis loads over 12 and 24 h, respectively (Fig. 4a). M. smegmatis-infected macrophage cells were treated with colistin, ceftazidime pentahydrate, ethambutol, or a combination of each permeabilizing drug and anti-*inhA* PNA for 24 h. The concentration for each permeabilizing drug was selected such that it was nontoxic to macrophage cells and only minimally affected intracellular bacterial survival, i.e., intracellular bacterial loads were not affected by more than 1 log-fold. Four mg/L of colistin in combination with 2.5 or 5 μM anti-*inhA* PNA enabled identical intracellular bacterial clearance to that of the corresponding free anti-*inhA* PNA treatment. This indicates that colistin does not have any effect, either beneficial or adverse, on the intracellular efficacy of PNA (Fig. 4b). Ceftazidime pentahydrate employed at a concentration of 128 mg/L improved the efficacy of 5 μM anti-*inhA* PNA, i.e., a 0.91 log-fold enhanced reduction in bacterial load compared to treatment with PNA alone (Fig. 4c). Additionally, 0.4 mg/L of ethambutol exhibited similar effects, where a combination of 0.4 mg/L ethambutol and 5 μM anti-*inhA* PNA achieved higher intracellular bacterial clearance (0.8 log-fold enhancement) than that achieved with PNA alone (Fig. 4d). Statistical significance determined through the Tukey-Kramer analysis of variance (ANOVA) *post hoc* test, however, indicated that even the combinations of ceftazidime pentahydrate or ethambutol with 5 μM anti-*inhA* PNA did not exhibit significance with 95% confidence. This is possibly due to the reduced accuracy at the detection limit of the drop-plating assay (35).

To investigate the efficacy of anti-*inhA* PNA and permeabilizing drug combinatorial treatments in tuberculosis therapy, similar experiments were carried out against M. tuberculosis H37Ra. For this purpose, we designed a PNA sequence complementary to the start site of the *inhA* gene in M. tuberculosis. The PNA was purchased as a PNA-CPP conjugate, where the CPP used was (KFF)_3_K.

### Efficacy of anti-*inhA* PNA, permeabilizing drugs, and their combinations against *M. tuberculosis* H37Ra.

**FIG 5:**
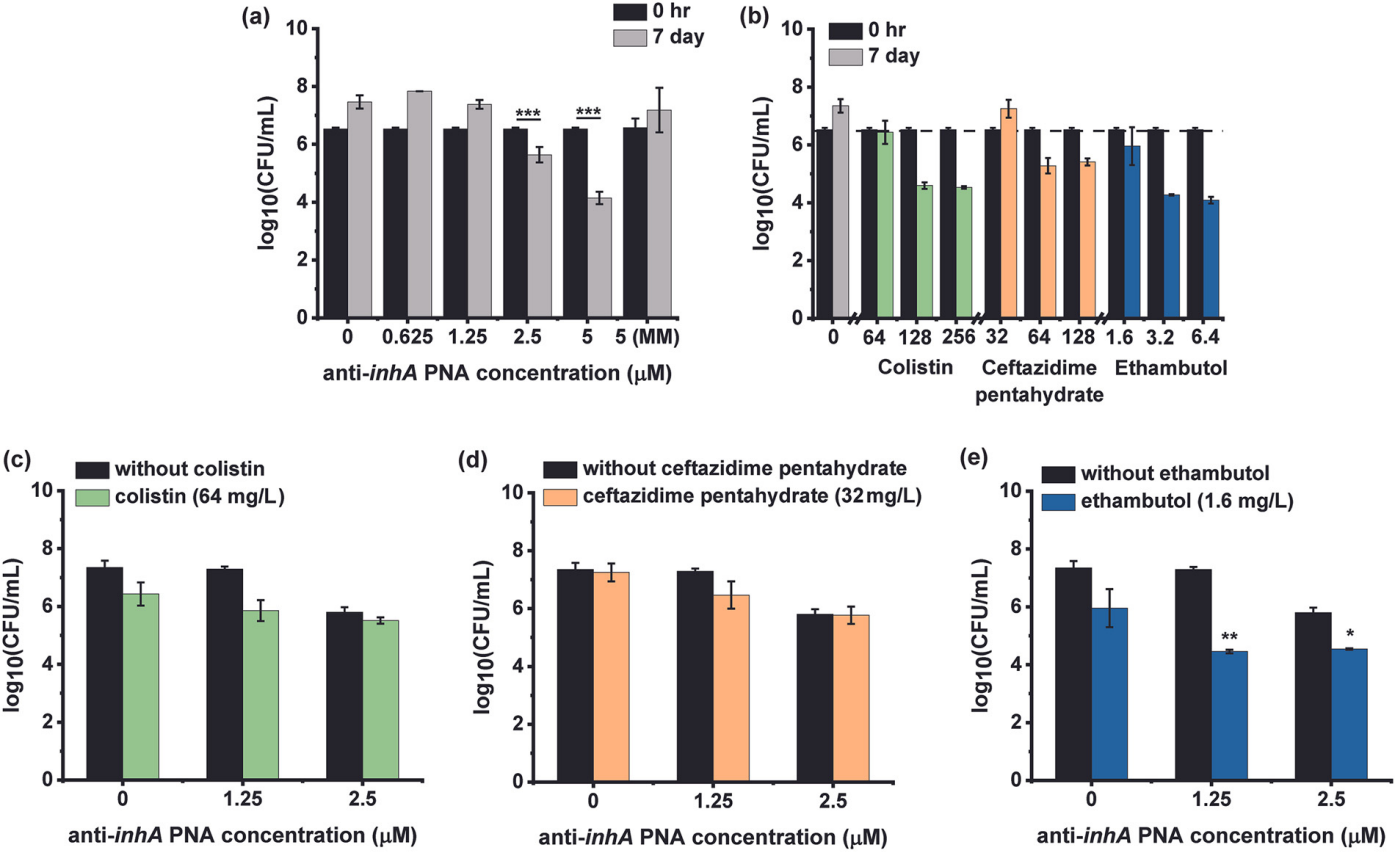
Effect of anti-*inhA* PNA and permeabilizing drugs alone and in combination against M. tuberculosis H37Ra treated for 7 days at *37°C a*nd 5% CO_2_. (a) Bacterial load before and after treatment with 0 to 5 μM anti-*inhA* PNA and 5 μM mismatch (MM) PNA (significance determined by Student's *t* test, where *** indicates *P* ≤ 0.001). (b) Bacterial load before and after treatment with 64 to 256 mg/L colistin, 32 to 128 mg/L ceftazidime pentahydrate, and 1.6 to 6.4 mg/L ethambutol. Dashed line marks the initial bacterial load for all samples. (c) Combinatorial effect of colisitn and anti-*inhA* PNA. (d) Combinatorial effect of ceftazidime pentahydrate and anti-*inhA* PNA. (e) Combinatorial effect of ethambutol and anti-*inhA* PNA. Temperature was maintained at 37°C with 5% CO_2_. Significance between combinatorial treatment and individual drug*/*PNA treatment, determined through the Tukey Kramer *post hoc* test, is indicated by * for *P* ≤ 0.05 or ** for *P* ≤  0.01. All data were obtained from 3 biological replicates and error bars indicate the standard deviation of each respective sample.

The antibacterial efficacy of anti-*inhA* PNA was screened against M. tuberculosis H37Ra over a range of concentrations. Low concentrations of 0.625 and 1.25 μM were found to be ineffective, while 2.5 and 5 μM anti-*inhA* PNA resulted in 1 and 2.4 log-fold reductions in bacterial load, respectively (Fig. 5a). A mismatch PNA sequence, having no binding site in the M. tuberculosis genome, was also tested as a control, and was found to have no effect on bacterial growth at a concentration of 5 μM (Fig. 5a). Prior to testing the combinatorial effects of permeabilizing drugs and PNA against M. tuberculosis, the MIC of each permeabilizing drug was determined after a 7-day treatment time. M. tuberculosis H37Ra growth inhibition was observed at 64 mg/L of colistin, 32 to 64 mg/L of ceftazidime pentahydrate, and 1.6 mg/L of ethambutol. These results were in correlation with previous reports showing the MICs of colistin, ceftazidime, and ethambutol to be 64 mg/L (25), 32 mg/L (26), and 2.5 to 5 mg/L (36), respectively, against M. tuberculosis. A combinatorial treatment of colistin or ceftazidime pentahydrate and anti-*inhA* PNA showed no overall benefit compared to the individual treatment with drug or PNA alone (Fig. 5c and d). This is likely due to a reduced ability of colistin and ceftazidime pentahydrate to permeabilize the cell membrane of M. tuberculosis H37Ra. Ethambutol in combination with 1.25 or 2.5 μM anti-*inhA* PNA, however, does improve the overall efficacy, causing a 2.8 or 1.3 log-fold enhanced reduction in bacterial load, respectively (Fig. 5e). Being an anti-TB agent itself, ethambutol effectively targets and permeates the cell wall of M. tuberculosis, enabling an enhanced efficacy in combinatorial treatment with anti-*inhA* PNA. Thus, similar to our observations for M. smegmatis, permeabilizing drugs can improve the efficacy of anti-*inhA* PNA against M. tuberculosis H37Ra. The efficacy of such a combinatorial therapy could be dependent on the permeabilizing potential of the drug.

### Effect of anti-*inhA* PNA and its combinations on the survival of intramacrophage *M. tuberculosis* H37Ra.

**FIG 6:**
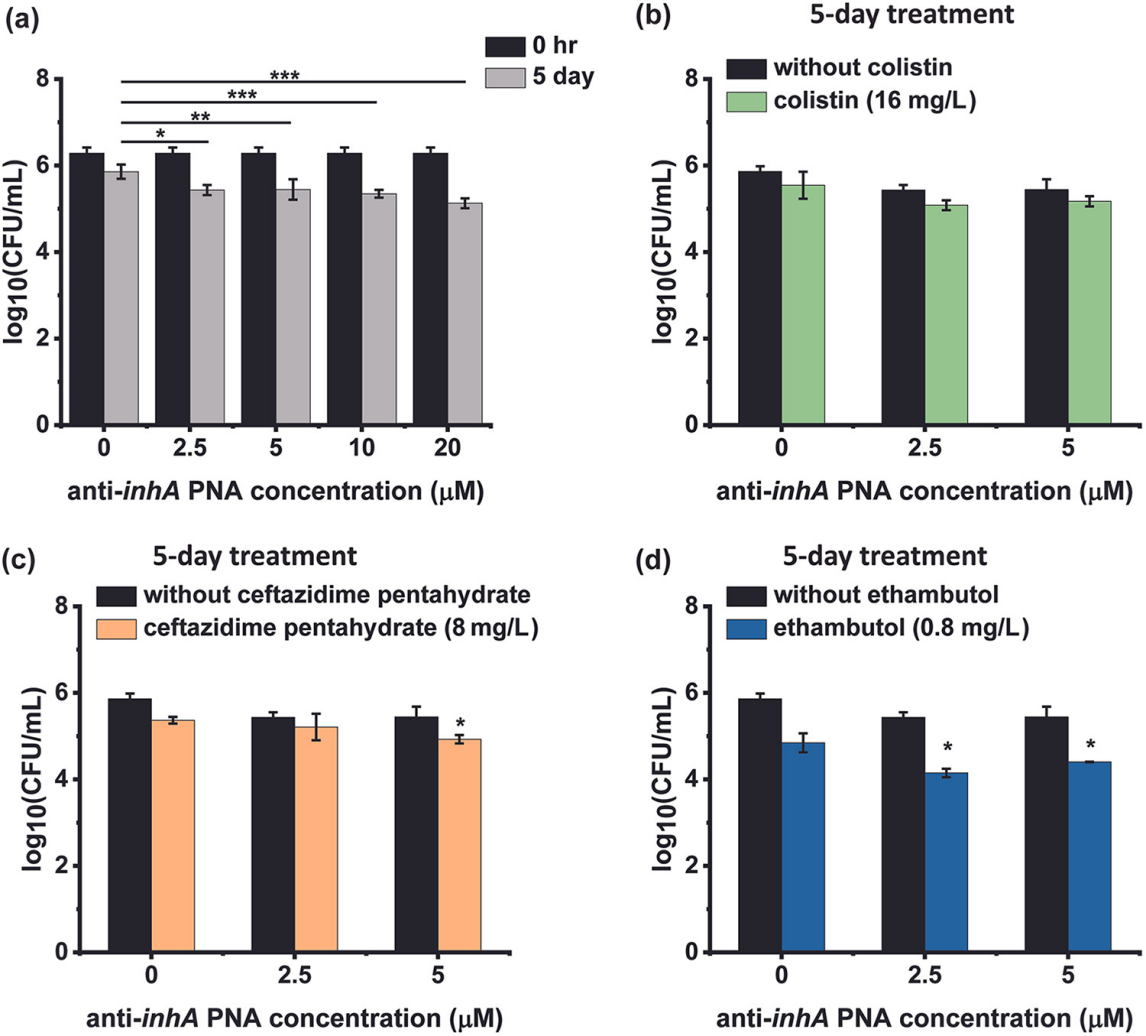
The survival of intramacrophage M. tuberculosis H37Ra upon treatment with anti-*inhA* PNA, permeabilizing drugs, and drug-PNA combinations. (a) Intracellular bacterial load at 0 h and 5 day after treatment with anti-*inhA* PNA at 37°C, 5% CO_2_. Intracellular bacterial load at 5 day after treatment with (b) colistin, (c) ceftazidime pentahydrate, or (d) ethambutol and their combination with anti-*inhA* PNA at 37°C, 5% CO_2_. The data were plotted from a sample size of *n* = 3, where error bars indicate the standard deviation of each sample. Significance of the combinatorial treatments with respect to individual drug and PNA treatments is denoted by *, *P* ≤ 0.05.

The efficacies of anti-*inhA* PNA and its combinations with permeabilizing drugs against intracellular M. tuberculosis were tested. It was observed that 2.5 μM anti-*inhA* PNA achieves effects similar to those observed for the untreated control, with higher PNA concentrations of 5, 10, and 20 μM enabling log reductions of 0.51, 0.55, and 0.75, respectively (Fig. 6a). Treatment with 16 mg/L colistin in combination with 2.5 or 5 μM anti-*inhA* has no effect on the efficacy of PNA (Fig. 6b). Treatment with 8 mg/L of ceftazidime pentahydrate improves the efficacy of 5 μM anti-*inhA* PNA, reducing the intracellular bacterial load by 1.2 log-fold (Fig. 6c). Treatment with 0.8 mg/L of ethambutol combined with 2.5 or 5 μM anti-*inhA* PNA showed 1.3 and 1.1 log-fold reductions in intracellular bacterial load, respectively (Fig. 6d). Thus, the combinatorial treatment achieves 7- to 20-times greater bacterial clearance than treatment with 2.5 to 5 μM anti-*inhA* PNA alone, respectively.

## DISCUSSION

Tuberculosis is the leading cause of deaths resulting from a single infectious agent after COVID-19, with the mortality rate having increased between 2019 and 2020 (37). The emergence of antimicrobial-resistant strains of M. tuberculosis further hinders treatment efforts and necessitates the development of improved therapies (37, 38). PNA, by targeting *inhA* in M. smegmatis, has anti-mycobacterial properties (20). However, its efficacy against intracellular bacteria and M. tuberculosis (the causative agent of tuberculosis) remained unknown. Furthermore, its antimicrobial efficacy was observed at concentrations of 5 μM and greater. Our study therefore aimed to investigate the therapeutic potential of anti-*inhA* PNA against M. smegmatis and M. tuberculosis, both in culture and intramacrophage. We also investigated a combinatorial treatment approach to potentiate PNA activity and reduce efficacious PNA concentrations.

This study confirmed that anti-*inhA* PNAs of 10 to 12 bp which target the start site of *inhA* in M. smegmatis or M. tuberculosis exhibit anti-mycobacterial properties. Anti-*inhA* PNA inhibited M. smegmatis growth at concentrations of 0.625 to 5 μM, while concentrations of 2.5 μM and greater exhibited bactericidal effects against M. tuberculosis. This anti-mycobacterial ability was attributed to its optimal length (6, 39, 40), CPP conjugation that promoted its uptake into the cell (5, 14, 41), and target start site-binding which enabled an efficient anti-sense effect (40). Low intracellular bacterial loads of M. smegmatis were partially cleared upon treatment with anti-*inhA* PNA, in a concentration- and time-dependent manner. However, concentrations as high as 20 μM only minutely affected intracellular M. tuberculosis survival. This reduced efficacy against intracellular bacteria could be attributed to compartmentalization of the internalized PNA making it less accessible to the intracellular bacteria (42). The overall high PNA concentration requirements for treatment could be due to the poor penetration capacity of PNA, since permeable mutants are more susceptible than wild-type (WT) strains to anti-sense PNA (7, 19). Hence, we hypothesized that combinatorial treatments with a permeabilizing drug and PNA could enable reductions in efficacious PNA concentrations.

Combining anti-*inhA* PNA with permeabilizing drugs enhances its antibacterial efficacy, with combinations with ethambutol showing the most promise. This corresponded with the highest increase in permeability observed when M. smegmatis cells were treated with ethambutol. Further, our study also confirmed an improved anti-*inhA* PNA efficacy against intracellular M. smegmatis/M. tuberculosis when it was combined with ethambutol. Studies by Dryselius et al. (2005) and Pantenge et al. (2013) reported that combinations of PNA and antibiotics inhibiting the same target through mRNA and protein inhibition result in synergistic responses in E. coli, S. aureus, and Streptococcus pyogenes (43, 44). Thus, the addition of an anti-*inhA* PNA to the current anti-TB regimen, where first-line drugs include rifampicin, isoniazid, pyrazinamide, and ethambutol (45), could possibly improve therapeutic efficacies. The presence of ethambutol in the existing treatment would also enable a low-concentration requirement of PNA for achieving desirable efficacy.

When testing permeabilizing drugs in combination with INH, an anti-TB drug targeting the same mycolic acid synthesis step, INH exhibited enhanced efficacy only in combination with ceftazidime pentahydrate against M. smegmatis. However, colistin and ethambutol were shown to have synergistic responses with INH against M. tuberculosis (25, 46). This difference in combinatorial efficacy could be attributed to differences between the M. smegmatis and M. tuberculosis strains in the cell wall (47) and the presence of regulatory protein (believed to be involved in synergy between ethambutol and INH in M. tuberculosis) (46). These results also deviated from those of our permeabilizing drug-PNA combinatorial studies, which may be due to the inability of colistin and ethambutol to sufficiently increase intracellular INH concentrations to the levels required to achieve a visible change in INH efficacy. In contrast, PNAs are known to be resistant to efflux mechanisms (7, 48) and hence their accumulation in the cell is only affected by factors which affect its influx. We showed that these permeabilizing drugs, at their inhibitory concentrations, facilitate the entry of substances into the cell. Thus, we believe that colistin, ceftazidime pentahydrate, and ethambutol potentiated the efficacy of anti-*inhA* PNA by promoting its entry into the mycobacterial cell.

The anti-*inhA* PNA designed against M. tuberculosis is also hypothesized to overcome isoniazid resistance, since isoniazid resistance has been demonstrated to be primarily through mutations in the *katG* gene (33). Furthermore, the anti-*inhA* PNA is designed to bind to the −5 bp to +7 bp region in *inhA.* This region of the *inhA* promoter in M. tuberculosis reports no mutation frequency among resistant species (49). This anti-*inhA* PNA could, therefore, also have the potential to overcome drug resistance.

In conclusion, combinatorial therapies involving the use of essential gene-targeting PNA and permeabilizing drugs like ethambutol present a new avenue of therapy for diseases such as tuberculosis which urgently need more effective antibacterial treatment approaches. The permeabilizing drugs increase cell wall permeability and, in turn, possibly enable the uptake of the anti-*inhA* PNA. This allows enhanced efficacy of the anti-*inhA* PNA such that its effective concentrations can also be reduced. Furthermore, such a system is effective against both extracellular and intramacrophage M. smegmatis and M. tuberculosis H37Ra. It is also our belief that the anti-*inhA* PNA designed against M. tuberculosis will continue to show promise against drug-resistant strains of M. tuberculosis.

## MATERIALS AND METHODS

### Bacterial strains and culturing.

The bacterial strains used in this study, Mycobacterium smegmatis mc^2^ 155 and Mycobacterium tuberculosis H37Ra, were obtained from Jaya Tyagi at the All India Institute of Medical Sciences, Delhi, and from Vinay Nandicoori at National Institute of Immunology, Delhi, respectively. M. smegmatis was cultured to an OD_600_ (optical density at 600 nm) of 0.5 in Middlebrook 7H9 (HiMedia) broth, supplemented with 0.44% glycerol (Rankem) and 0.15% Tween 80 (HiMedia), at 37°C and 200 rpm. M. tuberculosis H37Ra, cultured in a biosafety level 2+ facility, was grown in Middlebrook 7H9 medium, supplemented with 0.44% glycerol, 0.15% Tween 80, and 10% albumin-dextrose-NaCl solution (ADN), at 37°C and 180 rpm. The 0.5-OD_600_ cultures were used for testing the efficacy of compounds, membrane permeability assays, and infection.

### Human cell line and culturing.

THP-1 cells were received from Vinay Nandicoori at the National Institute of Immunology (Delhi). THP-1 cells were passaged in complete RPMI medium [RPMI medium supplemented with 10% fetal bovine serum (FBS), HiMedia], penicillin (100 IU, Sigma-Aldrich), and streptomycin (100 mg/L, Sigma-Aldrich) and cultured at 37°C and 5% CO_2_. The THP-1 monocytes were differentiated to macrophages by treatment with 0.025 mg/L phorbol 12-myristate acetate (PMA, Sigma-Aldrich) for 24 h at 37°C and 5% CO_2_. Differentiated THP-1 cells were then cultured in fresh complete RPMI medium for 24 h prior to further treatment or infection.

### Peptide nucleic acid design.

The 10-bp anti-*inhA* PNA sequence was selected from the study by Kulyte et al. (20) and its target site was confirmed bioinformatically using the blastn (https://blast.ncbi.nlm.nih.gov/Blast.cgi) and SnapGene (v5.2, http://www.snapgene.com) tools. An anti-*inhA* PNA sequence was also designed to target the gene start site of M. tuberculosis H37Ra, M. tuberculosis H37Rv, and M. bovis. For experimentation, the PNAs were obtained from PANAGENE (Daejeon, Republic of Korea) as PNA-CPP (KFFKFFKFFK) conjugates.

### Efficacy testing using a broth microdilution assay.

Here, 100-μL aliquots of a 10-fold diluted, 0.5-OD_600_ M. smegmatis culture were treated with 0.625 to 5 μM anti-*inhA* PNA or permeabilizing drugs (4 to 16 mg/L colistin, 128 to 512 mg/L ceftazidime pentahydrate, or 0.8 to 3.2 mg/L ethambutol) for 48 h at 37°C. All three permeabilizing drugs were purchased from HiMedia. Post-treatment, the cultures were drop-plated to determine the drop count in CFU/mL (equation below). Briefly, 10-μL drops of serially diluted samples were distinctly loaded on a Luria Bertani (LB) agar (HiMedia) plate and incubated for 48 h at 37°C. The colony count and corresponding dilution factor were noted. The lowest concentration of antibacterial agent that resulted in no change in bacterial count compared to the initial cell count was determined as the MIC.

$$\text{Drop count }\left(\frac{\text{CFU}}{\text{mL}}\right)=\frac{\text{Colony count}\times \text{dilution factor}}{0.01}$$

The efficacies of anti-*inhA* PNA (0.625 to 5 μM) and the permeabilizing drugs [colistin (64 to 256 mg/L), ceftazidime pentahydrate (32 to 128 mg/L), and ethambutol (1.6 to 6.4 mg/L)] were tested against M. tuberculosis H37Ra over 5 days at 37°C and 5% CO_2_. Viable cell densities after treatment were determined through a drop plating assay carried out on Middlebrook 7H11 agar and incubated for 30 days.

Permeabilizing drug-PNA combination studies were carried out in an identical manner, combining the MIC of the permeabilizing drug with varied concentrations of PNA.

### Efficacy testing using the disc diffusion assay.

Briefly, 10^5^ cells of a 0.5 OD_600_ M. smegmatis culture was spread plated on to an LB agar plate. Sterile cellulose discs were distinctly placed on the agar plate and colistin, ceftazidime pentahydrate, ethambutol, or isoniazid (INH) were loaded on the discs as desired. The agar plates were incubated at 37°C for 48 h and the diameters of the clearance zones, observed after incubation, were measured.

### Membrane permeability.

The membrane permeability was monitored through an NPN (1-*N*-phenylnapthylamine, HiMedia) assay (50). Briefly, M. smegmatis cells were treated with 16 mg/L of colistin, 256 mg/L of ceftazidime pentahydrate, and 1.6 mg/L of ethambutol for 4 h at 37°C and 200 rpm. The 0.5-OD_600_ treated cells were pelleted out from each sample and resuspended in 1 mL sterile ice-cold 5 mM HEPES (HiMedia) buffer. A 100-μL volume of these cells was aliquoted in a sterile microcentrifuge tube and mixed with 100 μL of ice-cold 50 μM NPN prepared in HEPES buffer, These NPN-stained cells were immediately imaged in a laser scanning confocal microscope (LSM 780, Carl Zeiss) using a 63× apochromatic oil objective. The excitation wavelength used was 405 nm and the emission was detected using a photon multiplier tube detector in a range of 410 to 500 nm. Approximately 70 single cell images were further analyzed using ZenBlue and ImageJ softwares to determine the corrected total cell fluorescence.

### Toxicity.

Differentiated THP-1 cells were washed and acclimatized in fresh complete RPMI medium for 24 h. These cells were then treated with 32 to 128 mg/L of colistin,128 to 1024 mg/L of ceftazidime pentahydrate, and 4 to 16 mg/L of ethambutol for 24 and 48 h. Post-treatment, the cells were trypsinized and cell counts were quantified using a Trypan Blue (0.4%) solution for manual cell counting in a hemocytometer.

### Macrophage infection.

Differentiated THP-1 cells were infected with M. smegmatis/M. tuberculosis H37Ra at an MOI of 10:1, where the bacterial culture was prepared to 0.5 OD_600_ in complete RPMI medium after syringe treatment to break up bacterial clumps. The macrophages and mycobacteria were cocultured for 3 h at 37°C and 5% CO_2_, after which the extracellular bacteria were removed and washed off thrice with phosphate-buffered saline supplemented with Ca-Mg. Complete RPMI medium containing 200 μg/mL amikacin was added to each well after the final wash, and the cells were incubated for 4 h at 37°C and 5% CO_2_. This ensured the complete elimination of extracellular bacteria. The amikacin was then washed off. This is considered the 0-h time point of infection. Fresh complete RPMI medium was added to the treatment wells, while the macrophages in the representative wells were lysed using 0.05% SDS to determine the 0-h bacterial load. The infected cells were then treated with PNA (0.625 to 5 μM), colistin, ceftazidime pentahydrate, ethambutol, or selected combinations of a permeabilizing drug and PNA for 12 or 24 h (M. smegmatis infection) or for 5 days (M. tuberculosis H37Ra infection).

### Statistical Analysis.

Individual treatments with anti-*inhA* PNA or permeabilizing drugs were analyzed using the Student’s *t* test. Statistical significance for combinatorial treatment studies was determined through ANOVA followed by the Tukey-Kramer *post hoc* test.

## References

[1] Lee HT, Kim SK, Yoon JW. 2019. Antisense peptide nucleic acids as a potential anti-infective agent. J Microbiol 57:423–430. doi:10.1007/s12275-019-8635-4. 31054136

[2] Nielsen PE. 2001. Peptide nucleic acids as antibacterial agents via the antisense principle. Expert Opin Invest Drugs 10:331–341. doi:10.1517/13543784.10.2.331. 11178345

[3] Quijano E, Bahal R, Ricciardi A, Saltzman WM, Glazer PM. 2017. Therapeutic peptide nucleic acids: principles, limitations, and opportunities. Yale J Biol Med 90:583–598. 29259523 PMC5733847

[4] Narenji H, Teymournejad O, Rezaee MA, Taghizadeh S, Mehramuz B, Aghazadeh M, Asgharzadeh M, Madhi M, Gholizadeh P, Ganbarov K, Yousefi M, Pakravan A, Dal T, Ahmadi R, Samadi Kafil H. 2020. Antisense peptide nucleic acids against *ftsZ* and *efaA* genes inhibit growth and biofilm formation of *Enterococcus faecalis*. Microb Pathog 139:e103907. doi:10.1016/j.micpath.2019.103907. 31811888

[5] Eriksson M, Nielsen PE, Good L. 2002. Cell permeabilization and uptake of antisense peptide-peptide nucleic acid (PNA) into *Escherichia coli*. J Biol Chem 277:7144–7147. doi:10.1074/jbc.M106624200. 11739379

[6] Goltermann L, Yavari N, Zhang M, Ghosal A, Nielsen PE. 2019. PNA length restriction of antibacterial activity of peptide-PNA conjugates in *Escherichia coli* through effects of the inner membrane. Front Microbiol 10:1032. doi:10.3389/fmicb.2019.01032. 31178830 PMC6542938

[7] Good L, Sandberg R, Larsson O, Nielsen PE, Wahlestedt C. 2000. Antisense PNA effects in *Escherichia coli* are limited by the outer-membrane LPS layer. Microbiology (Reading) 146:2665–2670. doi:10.1099/00221287-146-10-2665. 11021941

[8] Kurupati P, Tan KS, Kumarasinghe G, Poh CL. 2007. Inhibition of gene expression and growth by antisense peptide nucleic acids in a multiresistant beta-lactamase-producing *Klebsiella pneumoniae* strain. Antimicrob Agents Chemother 51:805–811. doi:10.1128/AAC.00709-06. 17158940 PMC1803136

[9] Maekawa K, Azuma M, Okuno Y, Tsukamoto T, Nishiguchi K, Setsukinai K, Maki H, Numata Y, Takemoto H, Rokushima M. 2015. Antisense peptide nucleic acid-peptide conjugates for functional analyses of genes in *Pseudomonas aeruginosa*. Bioorg Med Chem 23:7234–7239. doi:10.1016/j.bmc.2015.10.020. 26602085

[10] Goh S, Boberek JM, Nakashima N, Stach J, Good L. 2009. Concurrent growth rate and transcript analyses reveal essential gene stringency in *Escherichia coli*. PLoS One 4:e6061. doi:10.1371/journal.pone.0006061. 19557168 PMC2698124

[11] Mondhe M, Chessher A, Goh S, Good L, Stach JE. 2014. Species-selective killing of bacteria by antimicrobial peptide-PNAs. PLoS One 9:e89082. doi:10.1371/journal.pone.0089082. 24558473 PMC3928365

[12] Nekhotiaeva N, Awasthi SK, Nielsen PE, Good L. 2004. Inhibition of *Staphylococcus aureus* gene expression and growth using antisense peptide nucleic acids. Mol Ther 10:652–659. doi:10.1016/j.ymthe.2004.07.006. 15451449

[13] Rajasekaran P, Alexander JC, Seleem MN, Jain N, Sriranganathan N, Wattam AR, Setubal JC, Boyle SM. 2013. Peptide nucleic acids inhibit growth of *Brucella suis* in pure culture and in infected murine macrophages. Int J Antimicrob Agents 41:358–362. doi:10.1016/j.ijantimicag.2012.11.017. 23305655 PMC3834731

[14] Good L, Awasthi SK, Dryselius R, Larsson O, Nielsen PE. 2001. Bactericidal antisense effects of peptide-PNA conjugates. Nat Biotechnol 19:360–364. doi:10.1038/86753. 11283595

[15] Montazersaheb S, Hejazi MS, Nozad Charoudeh H. 2018. Potential of peptide nucleic acids in future therapeutic applications. Adv Pharm Bull 8:551–563. doi:10.15171/apb.2018.064. 30607328 PMC6311635

[16] Wojciechowska M, Rownicki M, Mieczkowski A, Miszkiewicz J, Trylska J. 2020. Antibacterial peptide nucleic acids: facts and perspectives. Molecules 25:559. doi:10.3390/molecules25030559. PMC7038079 32012929

[17] Oyaghire SN, Quijano E, Piotrowski-Daspit AS, Saltzman WM, Glazer PM. 2020. Poly(lactic-co-glycolic acid) nanoparticle delivery of peptide nucleic acids *in vivo*. Methods Mol Biol 2105:261–281. doi:10.1007/978-1-0716-0243-0_17. 32088877 PMC7199467

[18] Hansen AM, Bonke G, Larsen CJ, Yavari N, Nielsen PE, Franzyk H. 2016. Antibacterial peptide nucleic acid-antimicrobial peptide (PNA-AMP) conjugates: antisense targeting of fatty acid biosynthesis. Bioconjug Chem 27:863–867. doi:10.1021/acs.bioconjchem.6b00013. 26938833

[19] Tan XX, Actor JK, Chen Y. 2005. Peptide nucleic acid antisense oligomer as a therapeutic strategy against bacterial infection: proof of principle using mouse intraperitoneal infection. Antimicrob Agents Chemother 49:3203–3207. doi:10.1128/AAC.49.8.3203-3207.2005. 16048926 PMC1196239

[20] Kulyte A, Nekhotiaeva N, Awasthi SK, Good L. 2005. Inhibition of *Mycobacterium smegmatis* gene expression and growth using antisense peptide nucleic acids. J Mol Microbiol Biotechnol 9:101–109. doi:10.1159/000088840. 16319499

[21] Bialvaei AZ, Samadi Kafil H. 2015. Colistin, mechanisms and prevalence of resistance. Curr Med Res Opin 31:707–721. doi:10.1185/03007995.2015.1018989. 25697677

[22] Hayes MV, Orr DC. 1983. Mode of action of ceftazidime: affinity for the penicillin-binding proteins of *Escherichia coli* K12, *Pseudomonas aeruginosa* and *Staphylococcus aureus*. J Antimicrob Chemother 12:119–126. doi:10.1093/jac/12.2.119. 6413485

[23] Goude R, Amin AG, Chatterjee D, Parish T. 2009. The arabinosyltransferase EmbC is inhibited by ethambutol in *Mycobacterium tuberculosis*. Antimicrob Agents Chemother 53:4138–4146. doi:10.1128/AAC.00162-09. 19596878 PMC2764220

[24] Korycka-Machala M, Rumijowska-Galewicz A, Dziadek J. 2005. The effect of ethambutol on mycobaterial cell wall permeability to hydrophobic compounds. Polish J Microbiology 54:5–11. 16209089

[25] Bax HI, de Steenwinkel JE, Ten Kate MT, van der Meijden A, Verbon A, Bakker-Woudenberg IA. 2015. Colistin as a potentiator of anti-TB drug activity against *Mycobacterium tuberculosis*. J Antimicrob Chemother 70:2828–2837. doi:10.1093/jac/dkv194. 26183185

[26] Deshpande D, Srivastava S, Chapagain ML, Lee PS, Cirrincione KN, Pasipanodya JG, Gumbo T. 2017. The discovery of ceftazidime/avibactam as an anti-*Mycobacterium avium* agent. J Antimicrob Chemother 72:i36–i42. doi:10.1093/jac/dkx306. 28922808

[27] Bai H, Sang G, You Y, Xue X, Zhou Y, Hou Z, Meng J, Luo X. 2012. Targeting RNA polymerase primary sigma70 as a therapeutic strategy against methicillin-resistant *Staphylococcus aureus* by antisense peptide nucleic acid. PLoS One 7:e29886. doi:10.1371/journal.pone.0029886. 22253815 PMC3254626

[28] Soofi MA, Seleem MN. 2012. Targeting essential genes in *Salmonella enterica* serovar Typhimurium with antisense peptide nucleic acid. Antimicrob Agents Chemother 56:6407–6409. doi:10.1128/AAC.01437-12. 23006748 PMC3497187

[29] Lee HT, Kim SK, Lee JB, Yoon JW. 2019. A novel peptide nucleic acid against the cytidine monophosphate kinase of *S. aureus* inhibits staphylococcal infection *in vivo*. Mol Ther Nucleic Acids 18:245–252. doi:10.1016/j.omtn.2019.08.021. 31581048 PMC6796767

[30] Chen W, Dong B, Liu W, Liu Z. 2020. Recent advances in peptide nucleic acids as antibacterial agents. Curr Med Chem 28:1104–1125. doi:10.2174/0929867327666200602132504. 32484766

[31] Marrakchi H, Laneelle G, Quemard AK. 2000. InhA, a target of the anti-tuberculous drug isoniazid, is involved in a mycobacterial fatty acid elongation system, FAS-II. Microbiology 146:289–296. doi:10.1099/00221287-146-2-289. 10708367

[32] Kaur P, Agarwal S, Datta S. 2009. Delineating bacteriostatic and bactericidal targets in mycobacteria using IPTG inducible antisense expression. PLoS One 4:e5923. doi:10.1371/journal.pone.0005923. 19526063 PMC2691988

[33] Manjunatha UH, S Rao SP, Kondreddi RR, Noble CG, Camacho LR, Tan BH, Ng SH, Ng PS, Ma NL, Lakshminarayana SB, Herve M, Barnes SW, Yu W, Kuhen K, Blasco F, Beer D, Walker JR, Tonge PJ, Glynne R, Smith PW, Diagana TT. 2015. Direct inhibitors of InhA are active against *Mycobacterium tuberculosis*. Sci Transl Med 7:269ra3. 10.1126/scitranslmed.3010597 PMC4383039 25568071

[34] Halder S, Yadav KK, Sarkar R, Mukherjee S, Saha P, Haldar S, Karmakar S, Sen T. 2015. Alteration of Zeta potential and membrane permeability in bacteria: a study with cationic agents. Springerplus 4:672. doi:10.1186/s40064-015-1476-7. 26558175 PMC4633473

[35] Jongenburger I, Reij MW, Boer EP, Gorris LG, Zwietering MH. 2010. Factors influencing the accuracy of the plating method used to enumerate low numbers of viable micro-organisms in food. Int J Food Microbiol 143:32–40. doi:10.1016/j.ijfoodmicro.2010.07.025. 20724016

[36] Koga H, Miyamoto J, Ohno H, Ogawa K, Tomono K, Tashiro T, Kohno S. 1997. A rapid drug susceptibility test for *Mycobacterium tuberculosis* using the hybridization protection assay. J Antimicrob Chemother 40:189–194. doi:10.1093/jac/40.2.189. 9301983

[37] Chakaya J, Khan M, Ntoumi F, Aklillu E, Fatima R, Mwaba P, Kapata N, Mfinanga S, Hasnain SE, Katoto P, Bulabula ANH, Sam-Agudu NA, Nachega JB, Tiberi S, McHugh TD, Abubakar I, Zumla A. 2021. Global Tuberculosis Report 2020: reflections on the global TB burden, treatment and prevention efforts. Int J Infect Dis 113 Suppl 1:S7–S12. doi:10.1016/j.ijid.2021.02.107. 33716195 PMC8433257

[38] Hu Y, Zheng X, Davies Forsman L, Ning Z, Chen C, Gao Y, Zhang Z, Lu W, Werngren J, Bruchfeld J, Hoffner S, Xu B. 2021. Emergence of additional drug resistance during treatment of multidrug-resistant tuberculosis in China: a prospective cohort study. Clin Microbiol Infect 27:1805–1813. doi:10.1016/j.cmi.2021.04.001. 33895338

[39] Ghosal A, Nielsen PE. 2012. Potent antibacterial antisense peptide-peptide nucleic acid conjugates against *Pseudomonas aeruginosa*. Nucleic Acids Ther 22:323–334. doi:10.1089/nat.2012.0370. PMC3464458 23030590

[40] Dryselius R, Aswasti SK, Rajarao GK, Nielsen PE, Good L. 2003. The translation start codon region is sensitive to antisense PNA inhibition in *Escherichia coli*. Oligonucleotides 13:427–433. doi:10.1089/154545703322860753. 15025910

[41] Koppelhus U, Awasthi SK, Zachar V, Holst HU, Ebbesen P, Nielsen PE. 2002. Cell-dependent differential cellular uptake of PNA, peptides, and PNA-peptide conjugates. Antisense Nucleic Acids Drug Dev 12:51–63. doi:10.1089/108729002760070795. 12074365

[42] Shiraishi T, Nielsen PE. 2014. Cellular delivery of peptide nucleic acids (PNAs). Methods Mol Biol 1050:193–205. doi:10.1007/978-1-62703-553-8_16. 24297361

[43] Dryselius R, Nekhotiaeva N, Good L. 2005. Antimicrobial synergy between mRNA- and protein-level inhibitors. J Antimicrob Chemother 56:97–103. doi:10.1093/jac/dki173. 15914490

[44] Patenge N, Pappesch R, Krawack F, Walda C, Mraheil MA, Jacob A, Hain T, Kreikemeyer B. 2013. Inhibition of Growth and Gene Expression by PNA-peptide Conjugates in *Streptococcus pyogenes*. Mol Ther Nucleic Acids 2:e132. doi:10.1038/mtna.2013.62. 24193033 PMC3889189

[45] World Health Organization (WHO). 2010. Standard treatment regimens. *In* Treatment of tuberculosis: guidelines, 4th ed. World Health Organization, Geneva, Switzerland.

[46] Zhu C, Liu Y, Hu L, Yang M, He ZG. 2018. Molecular mechanism of the synergistic activity of ethambutol and isoniazid against *Mycobacterium tuberculosis*. J Biol Chem 293:16741–16750. doi:10.1074/jbc.RA118.002693. 30185616 PMC6204910

[47] Yamada H, Yamaguchi M, Igarashi Y, Chikamatsu K, Aono A, Murase Y, Morishige Y, Takaki A, Chibana H, Mitarai S. 2018. *Mycolicibacterium smegmatis*, basonym *Mycobacterium smegmatis*, expresses morphological phenotypes much more similar to *Escherichia coli* than *Mycobacterium tuberculosis* in quantitative structome analysis and CryoTEM examination. Front Microbiol 9:1992. doi:10.3389/fmicb.2018.01992. 30258411 PMC6145149

[48] Nikravesh A, Dryselius R, Faridani OR, Goh S, Sadeghizadeh M, Behmanesh M, Ganyu A, Klok EJ, Zain R, Good L. 2007. Antisense PNA accumulates in *Escherichia coli* and mediates a long post-antibiotic effect. Mol Ther 15:1537–1542. doi:10.1038/sj.mt.6300209. 17534267

[49] Seifert M, Catanzaro D, Catanzaro A, Rodwell TC. 2015. Genetic mutations associated with isoniazid resistance in *Mycobacterium tuberculosis*: a systematic review. PLoS One 10:e0119628. doi:10.1371/journal.pone.0119628. 25799046 PMC4370653

[50] Padwal P, Bandyopadhyaya R, Mehra S. 2015. Biocompatible citric acid-coated iron oxide nanoparticles to enhance the activity of first-line anti-TB drugs in *Mycobacterium smegmatis*. J Chem Technol Biotechnol 90:1773–1781. doi:10.1002/jctb.4766.

